# Probiotics, Symptoms, and Gut Microbiota: What Are the Relations? A Randomized Controlled Trial in Subjects with Irritable Bowel Syndrome

**DOI:** 10.1155/2012/214102

**Published:** 2012-07-31

**Authors:** Per G. Farup, Morten Jacobsen, Solveig C. Ligaarden, Knut Rudi

**Affiliations:** ^1^Department of Research, Innlandet Hospital Trust, 2819 Gjøvik, Norway; ^2^Unit for Applied Clinical Research, Department of Cancer Research and Molecular Medicine, Norwegian University of Science and Technology, 7491 Trondheim, Norway; ^3^Department of Medicine, Østfold Hospital Trust, 1603 Fredrikstad, Norway; ^4^Department of Medicine, Innlandet Hospital Trust, 2819 Gjøvik, Norway; ^5^Nofima Mat AS, 1431 Ås, Norway

## Abstract

*Introduction*. Knowledge of the mechanism of action of probiotics in subjects with irritable bowel syndrome (IBS) is imperfect. *Objective*. This trial aimed at discriminating between a direct effect on the gut wall and an indirect effect caused by modulation of the fecal microbiota. *Design*. Randomized, double-blind, crossover trial. *Material and Methods*. Patients with IBS were given one capsule of 10^10^ CFU *L. plantarum* MF 1298 or placebo once daily. Symptoms were registered (score 0–15) and feces collected at the end of each period. The gut microbiota was analyzed with 16S rRNA gene analyses and results reported as proportions of Bacteroides, Faecalibacterium, and Lachnospiraceae and Simpson's D diversity score. *Results*. Sixteen participants (11 women) with a mean age of 50 years (SD 11) were available for the analyses. Intake of *L. plantarum* MF 1298 was associated with a significant aggravation of symptoms, but neither intake of *L. plantarum* MF 1298 nor symptoms were associated with the composition of the fecal microbiota (*P* values >0.10). *Conclusions*. The trial indicates that the symptomatic aggravation related to intake of *L. plantarum* MF 1298 was a direct effect of the microbe on the gut wall and not caused by changes in the fecal microbiota.

## 1. Introduction

The gut microbiota influences metabolism, epithelial function, immunity, and inflammation in the intestine and has been related to development, maintenance, and treatment of various disorders. Irritable bowel syndrome (IBS), which is a common biopsychosocial disorder with unknown etiology, is a disorder which has been linked to disturbances of the gut microbiota [1–4]. Since no highly effective treatment is available and the disorder has been associated with disturbances in the fecal microbiota, probiotics have been studied in several clinical trials. The effectiveness, which seems to be species and strain specific, varies from marked improvement to aggravation of symptoms [5, 6]. Overall, there seems to be a small, but statistically significant beneficial effect of probiotics in subjects with IBS [7–9]. 

The mode of action of probiotics is in part unknown. Among several features of probiotics possibly relevant to IBS, both direct effects on the gut wall and indirect effects via restoration of a disturbed fecal or mucosal microbiota or blockage of the effect of pathogenic bacteria have been mentioned [10–13]. Whatever the mechanism, probiotics have complex influence on nuclear receptor signaling and inflammatory pathways [13, 14]. 

We recently published a randomized placebo-controlled crossover trial showing an unfavorable effect of a candidate probiotic, *Lactobacillus* (*L*.) *plantarum *MF 1298, in subjects with IBS [6]. After publishing the study, we have had the opportunity to examine the subjects' fecal microbiota. The aim of the study was to discriminate between a direct effect of *L. plantarum *MF 1298 on the gut wall and an indirect effect caused by modulation of the fecal microbiota.

## 2. Methods 

### 2.1. Participants

Participants were recruited from a hospital-based gastroenterology outpatient clinic and a private gastroenterological practice. Subjects of 18 to 75 years of age with IBS according to the Roma II criteria and symptoms at the last three months were eligible for inclusion. Subjects using antibiotics or laxatives five weeks prior to inclusion or during the trial were excluded. The previous publication gives further details [6]. 

### 2.2. Study Design

The study was a randomized double-blind, placebo-controlled, crossover trial with a one-week run-in period followed by randomization and two three-week treatment periods separated by a four-week washout period. Background characteristics were assessed at inclusion. IBS symptoms were recorded on diary cards every evening during the run-in period, during the last week of the washout period, and the last week of the two treatment periods. Fecal samples were collected at the end of the run-in period, the washout period, and the two treatment periods. Data were collected at the outpatient clinic at Innlandet Hospital Trust, Gjøvik.

Unit for Applied Clinical Research, Norwegian University of Science and Technology, Trondheim, Norway performed the computer-based randomization and Faun Pharma, Norway, provided packed and numbered capsules containing 10^10^ CFU live, freeze-dried *L. plantarum* MF 1298 or placebo according to the randomization list. The capsules looked identical and were prescribed to be taken with liquid once daily. The participants and health care providers were blinded until all data were collected and clean file was established. 

### 2.3. Assessments

Seven gastrointestinal symptoms were recorded. Abdominal pain/discomfort, urgency, and bloating were recorded as none, mild, moderate, or severe (score 0–3); stool frequency as number of stools per day; stool consistency according to Bristol stool form scale (score 1–7); straining and incomplete bowel movement as yes/no (score: 1 or 0). An IBS symptom score (score 0–15) was calculated as the sum of these seven scores after “normalization” of stool frequency and consistency to achieve low scores for normal bowel habits. The “normalization” was performed as follows: stool frequency: 0 stool/day = 1; 1–3 stools/day = 0; 4-5 stools/day = 1; ≥6 stools/day = 2; stool consistency: Bristol stool scale 3–5 = 0; Bristol stool scale 2 and 6 = 1; Bristol stool scale 1 and 7 = 2. 

After collection, fecal samples were frozen in Carey Blair medium (Oxoid Ltd, Basingstoke, Hampshire, UK) and stored at −20°C. DNA isolation and purification was carried out using an automated procedure with silica particles (Bioclone Inc., San Diego, CA) [15]. DNA from the fecal samples were 16S rRNA gene amplified and sequenced using the direct sequencing approach [16]. The direct sequencing spectra were aligned and processed with use of MATLAB (MathWorks, Natick, MA) [17]. 

Multivariate curve resolution-alternating least squares (MCR-ALS) was used as an iterative approach (algorithm) to find the matrices of concentration profiles and pure component spectra from the mixed sequence spectra. The algorithm firstly identifies rank, or dimensionality of the data. In the next step, the pure spectra and concentrations of these are identified, assuming closure of the data [17]. MCR-ALS was implemented in Unscrambler version 9.8 (Camo, Woodbridge, NJ).

For three patients, we also generated sequence libraries using deep 454 pyrosequencing analyses. The same 16S rRNA amplicon as described above was used. The pyrosequencing was done at the Norwegian High-Throughput Sequencing Centre (University of Oslo, Norway).

### 2.4. Outcomes

The outcomes were IBS symptom score and the composition of the fecal microbiota reported as the relative proportion of Bacteroides, Faecalibacterium, and Lachnospiraceae. The Simpson's D diversity scores were calculated as one minus the sum of the squares of the relative proportions of Bacteroides, Faecalibacterium, and Lachnospiraceae, later referred to as “common.” Three patients had in addition Simpson's D and Shannon's H diversity scores calculated based on the deep pyrosequencing data, later referred to as “alternative”. Four paired data sets were available for each subject: the first one at the end of the run-in period, the second at the end of the first treatment period, the third at the end of the washout period, and the fourth at the end of the last treatment period. “Treatment effect” was changes during active treatment minus changes during placebo treatment.

### 2.5. Statistical Methods

The analyses were performed with parametric and nonparametric tests depending on normability tested with Kolmogorov-Smirnov test. Multiple imputations for missing data were performed with a model including all principal variables. Available data were used for descriptive statistics and unadjusted analyses, and multivariable analyses were performed on 20 datasets imputed for missing values. Predictors for treatment effect on IBS symptom score were performed with multivariable linear regression analyses. PASW statistics 18 was used for the analyses, and two-sided *P*-values ≤0.05 were regarded as statistically significant. 

### 2.6. Ethics

All subjects gave written informed consent to participate. The study was performed in accordance with the Declaration of Helsinki, approved by the Regional Committee for Medical Research Ethics in Central Norway, and registered in ClinicalTrials.gov (number NCT00355810).

## 3. Results

Twenty-eight patients were included and sixteen (five males and eleven females) with a mean age of 50 (SD = 11) years, BMI 24 (SD = 3) kg/m^2^, and symptom duration of 31 (SD = 17) years were available for the analyses: one had constipation-predominant, nine alternating, and six diarrhea-predominant IBS. For details see the previous paper by SC Ligaarden et al. [6]. One patient had incompletely filled in symptom questionnaire at one visit, and four patients had one missing fecal sample at different visits. *L. plantarum *MF1298 was detected in all fecal samples at the end of the active treatment period.

Table 1 gives the IBS symptom score, the relative proportion of Bacteroides, Faecalibacterium, and Lachnospiraceae, and the diversity scores during the trial.  Table 2 gives the “treatment effect” on IBS symptom score and on the fecal microbiota. Only the effect on symptoms was statistically significant. Active treatment induced an increase in IBS symptom score of 1.57 (*P* = 0.04) compared with placebo.

Correlations (Spearman's rho) between IBS symptom score and proportions of Bacteroides, Faecalibacterium, and Lachnospiraceae were 0.18 (*P* = 0.16), −0.09 (*P* = 0.50), and −0.09 (*P* = 0.52), respectively, and correlations between IBS symptoms and common Simpson's D, alternative Simpson's D, and Shannon's H diversity scores were −0.04 (*P* = 0.76), 0.32 (*P* = 0.31), and 0.13 (*P* = 0.70), respectively. Neither were the correlations between changes in IBS symptom scores and changes in the microbiota and diversity of the microbiota from one measurement to the next statistically significant (data not shown).

Table 3 gives predictors for “treatment effect” on symptoms (linear regression analyses). Only treatment with *L. plantarum* MF1298, not the fecal microbiota, was associated with changes in symptoms. 

**Figure 1:**
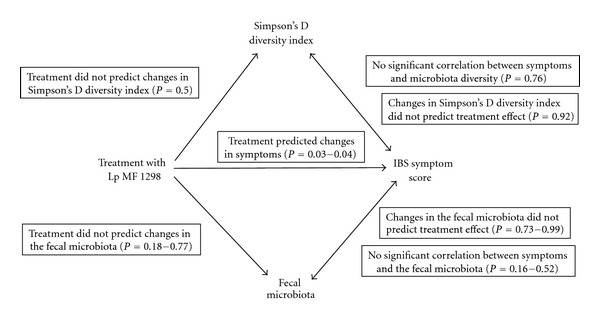
A schematic presentation of all results in the trial showing the associations between treatment with probiotics (Lp MF 1298), the fecal microbiota (composition and diversity), and symptoms.

Figure 1 shows a schematic overview of all results.

## 4. Discussion

In this study, the probiotic *L. plantarum* MF1298 had an unfavorable effect on symptoms, but no effect on the fecal microbiota, neither on the proportions of the bacteria nor on an overall diversity score. Intake of *L. plantarum* MF1298 predicted changes in symptoms, whereas changes in the proportions of the bacteria or changes in bacterial diversity did not. Even though the study could not exclude subtle changes in the microbiota or changes in the mucosal adherent flora, the symptomatic effect related to intake of *L. plantarum* MF1298 was most likely a direct effect of the bacterium on the gut wall and not an indirect effect mediated via changes in the gut microbiota.

 Reviews provide evidence for both a direct effect of probiotics on the gastrointestinal function, an indirect effect by modifying the gut microbiota which alters the gastrointestinal function, and a combination of the two [9–12]. But most studies report immunological and clinical effects without distinguishing between these modes of action.

Direct effects of probiotics have been substantiated in experimental models. *Lactobacillus plantarum* has been reported to prevent upregulation of adhesion molecules, improve histological inflammation, ameliorate colonic epithelial barrier dysfunction, prevent bacterial translocation, and reduce proinflammatory cytokine production in Il-10 knockout mice with spontaneous colitis [18, 19]. Reviews describe relations between probiotics, nuclear receptor signaling, and anti-inflammatory pathways and give summaries of published molecular mechanisms of probiotics [13, 14]. The complex signaling, immunological, and inflammatory processes, individual diversity, and species- and strain-specific actions of probiotics make the research challenging. *L. plantarum* MF1298 has direct effects on the gut *in vitro*. The strain adheres to the human colon adenoma cell line CaCo2, strengthens transepithelial resistance of a CaCo2 cell layer, and increases production of certain tight-junction proteins [20, 21]. These *in vitro* experiments with *L. plantarum* MF1298 support the finding that the effect most likely was a direct effect on the gut wall. 

 Differences between fecal and mucosal bacterial communities, between subjects with IBS and healthy persons and between subgroups of IBS have been described in several studies [1–4, 22, 23]. The findings have, however, not been consistent and reproducible between the studies, and it is unclear whether the differences between IBS and healthy subjects are primary or secondary [10]. Restoration of an altered gut microbiota has an effect on symptoms and is an attractive target for treatment of IBS [9, 12, 24]. Probiotics have prerequisites for restoration of gut microbiota by, for example, acidification of the colon by altering the fermentation, antimicrobial effects and blocking the action of pathogenic bacteria. Gut microbiota shifts toward that of healthy subjects have been reported after use of probiotics in subjects with IBS [25, 26].

*L. plantarum* MF1298 has an antimicrobial activity *in vitro* that could change the bacterial fecal composition, but too short treatment periods might have unrevealed the effect [21]. The lack of association between changes in symptoms and the gut microbiota in this trial, where the changes in symptoms were probably due to a direct effect of the probiotic on the gut wall, does not exclude that spontaneous fluctuations in symptoms are associated with changes in the gut microbiota.

The gut microbiota has been reported to differ between subjects with and without IBS [1–3]. This study allowed no comparison with healthy subjects, and no study with the same method and classification of the microbiota is available for comparisons. 

### 4.1. Strengths and Weaknesses

Classification of the complex fecal microbiota into only three groups renders detection of changes within each group impossible. A more detailed description of the microbiota with analyses of subtle changes would have been preferable. But since changes in large bacterial groups have been reported in subjects with IBS, the findings are relevant [2]. 

The fecal part of the microbiota, which was assessed in this trial, differs from the mucosal microbiota, which might be more important for the GI-function and symptoms. Analyses of the mucosal microbiota were not performed and could have given valuable additional information. However, derangement of the fecal microbiota in subjects with IBS and normalization after probiotics treatment have been reported [1, 2, 25, 26]. The detection of *L. plantarum *MF1298 in all fecal samples at the end of the active treatment period strengthened the study.

The treatment periods were long enough to provoke symptoms, but might have been too short to induce alterations in the microbiota. The fast-appearing symptomatic effect without demonstrable effect on the microbiota made an indirect effect mediated via changes in the microbiota unlikely. 

Small trials, like this one, increase the probability of type II errors.

## 5. Conclusion

The trial did not show any associations between the unfavorable symptomatic effect of *L. plantarum* MF1298, a candidate probiotic for IBS, and changes in the fecal microbiota. Although the methods were unsuitable for the detection of subtle changes in the microbiota or changes in the mucosal adherent flora, the unfavorable effect of *L. plantarum* MF1298 was most likely a direct effect on the gut wall and not an indirect effect mediated via changes in the fecal microbiota. In order to increase the understanding of the effect of probiotics and develop new and efficient ones, further research should call more attention to differentiation between the direct and indirect effects.

## Figures and Tables

**Table 1:** IBS symptom scores, the relative proportion of the components of the fecal microbiota, and the diversity indexes in the four parts of the trial. Results are given as mean (SD).

Variables	Run-in period	1st treatment period	Washout period	2nd treatment period
IBS symptom score (range 0–15)				
Active treatment 1st period	6.17 (1.07)	6.18 (1.83)	6.58 (2.55)	5.14 (2.12)
Placebo treatment 1st period	6.26 (2.20)	5.61 (1.31)	5.80 (2.61)	6.77 (1.85)
Bacteroides				
Active treatment 1st period	0.07 (0.05)	0.07 (0.06)	0.07 (0.07)	0.12 (0.10)
Placebo treatment 1st period	0.19 (0.17)	0.21 (0.16)	0.16 (0.12)	0.14 (0.10)
Faecalibacterium				
Active treatment 1st period	0.54 (0.23)	0.53 (0.21)	0.47 (0.26)	0.46 (0.26)
Placebo treatment 1st period	0.44 (0.23)	0.56 (0.21)	0.54 (0.15)	0.61 (0.25)
Lachnospiraceae				
Active treatment 1st period	0.38 (0.22)	0.40 (0.17)	0.46 (0.23)	0.42 (0.24)
Placebo treatment 1st period	0.37 (0.21)	0.23 (0.14)	0.30 (0.16)	0.26 (0.18)
“Common” Simpson's D				
Active treatment 1st period	0.46 (0.09)	0.48 (0.10)	0.46 (0.13)	0.48 (0.11)
Placebo treatment 1st period	0.52 (0.20)	0.51 (0.23)	0.54 (0.12)	0.46 (0.18)
“Alternative” Simpson's D				
Active treatment 1st period	321 (382)	59 (19)	67 (21)	28 (10)
Placebo treatment 1st period	92	135	33	89
“Alternative” Shannon's H				
Active treatment 1st period	6.42 (0.80)	5.86 (0.03)	5.73 (0.27)	5.47 (0.36)
Placebo treatment 1st period	6.09	6.42	5.79	6.12

**Table 2:** “Treatment effect” (increase during active treatment minus increase during placebo treatment) on symptoms, proportions of the fecal microbiota, and on fecal diversity.

Variables	Mean	95% CI of the mean	Statistics (*P* values)
IBS symptom score	1.57	0.10; 3.05	*P* = 0.04
Bacteroides	−0.01	−0.11; 0.08	ns (*P* = 0.77)
Faecalibacterium	−0.03	−0.18; 0.12	ns (*P* = 0.63)
Lachnospiraceae	0.05	−0.03; 0.13	ns (*P* = 0.18)
“Common” Simpson's D	−0.04	−0.15; 0.08	ns (*P* = 0.50)

**Table 3:** Predictors for “treatment effect” (increase in symptoms during active treatment minus increase during placebo treatment).

Independent variables	Statistics	
	*β*	*P* values
Active treatment	3.03–3.20	*P* = 0.029–0.035
Bacteroides	1.59	ns (*P* = 0.74)
Faecalibacterium	−0.02	ns (*P* = 0.99)
Lachnospiraceae	−1.39	ns (*P* = 0.73)
“Common” Simpson's D	−0.39	ns (*P* = 0.92)

The table gives the results of four linear regression analyses. “Treatment effect” is a dependent variable, and treatment is an independent variable in all analyses; changes in Simpson's diversity score and the proportions of Bacteroides, Faecalibacterium, and Lachnospiraceae are independent variables in each of the four analyses.
